# Extensive neuroadaptive changes in cortical gene-transcript expressions of the glutamate system in response to repeated intermittent MDMA administration in adolescent rats

**DOI:** 10.1186/1471-2202-9-39

**Published:** 2008-04-17

**Authors:** Anna MS Kindlundh-Högberg, Anna Blomqvist, Rana Malki, Helgi B Schiöth

**Affiliations:** 1 Department of Neuroscience, Division of Functional Pharmacology, Uppsala University, 751 24 Uppsala, Sweden

## Abstract

**Background:**

Many studies have focused on the implication of the serotonin and dopamine systems in neuroadaptive responses to the recreational drug 3,4-methylenedioxy-metamphetamine (MDMA). Less attention has been given to the major excitatory neurotransmitter glutamate known to be implicated in schizophrenia and drug addiction. The aim of the present study was to investigate the effect of repeated intermittent MDMA administration upon gene-transcript expression of the glutamate transporters (EAAT1, EAAT2-1, EAAT2-2), the glutamate receptor subunits of AMPA (GluR1, GluR2, GluR3), the glutamate receptor subunits of NMDA (NR1, NR2A and NR2B), as well as metabotropic glutamate receptors (mGluR1, mGluR2, mGluR3, mGluR5) in six different brain regions. Adolescent male Sprague Dawley rats received MDMA at the doses of 3 × 1 and 3 × 5 mg/kg/day, or 3× vehicle 3 hours apart, every 7^th ^day for 4 weeks. The gene-transcript levels were assessed using real-time PCR validated with a range of housekeeping genes.

**Results:**

The findings showed pronounced enhancements in gene-transcript expression of GluR2, mGluR1, mGluR5, NR1, NR2A, NR2B, EAAT1, and EAAT2-2 in the cortex at bregma +1.6. In the caudate putamen, mRNA levels of GluR3, NR2A, and NR2B receptor subunits were significantly increased. In contrast, the gene-transcript expression of GluR1 was reduced in the hippocampus. In the hypothalamus, there was a significant increase of GluR1, GluR3, mGluR1, and mGluR3 gene-transcript expressions.

**Conclusion:**

Repeated intermittent MDMA administration induces neuroadaptive changes in gene-transcript expressions of glutamatergic NMDA and AMPA receptor subunits, metabotropic receptors and transporters in regions of the brain regulating reward-related associative learning, cognition, and memory and neuro-endocrine functions.

## Background

The recreational drug 3,4-methylenedioxy-metamphetamine (MDMA; ecstasy) is popular among young people due to its energy and mood enhancing properties [1]. MDMA abuse is also associated with psychiatric side effect such as hallucinations, depression, reduced cognitive performance and memory loss [2-6]. The various actions and effects of MDMA have resulted in contradictive views among researchers regarding the degree of the harmfulness of the drug [7]. Many studies have focused on neuroadaptive responses in the serotonin and dopamine systems towards MDMA [8,9]. However, in order to achieve a comprehensive picture of neurobiochemical mechanisms underlying MDMA induced psychiatric changes, research on MDMA to a higher extent also has to be extended to include closely interacting systems.

The major excitatory neurotransmitter in the CNS is the glutamate system [10]. It is interactive with GABA, dopamine and serotonin, especially through the regulation of the corticofugal neuronal activity between the forebrain cortex and subcortical brain regions [11-15]. In addition to being implicated in learning plasticity and neural cell death [16], the glutamate system is implicated in addictive diseases to several drugs of abuse [14,17-19], as well as psychiatric conditions such as e.g. schizophrenia [13,14]. Nevertheless, only a few studies have been performed on MDMA and glutamate. Microiontophoretic application of MDMA has been shown to inhibit glutamate evoked firing of most cells in the nucleus accumbens [20], possibly through the drug mediated release of dopamine and serotonin [21]. The glutamatergic NMDA antagonist MK801 has been shown to protect against MDMA caused serotonin depletion and tryptophanehydroxylase decrease [22,23]. Moreover, ACEA (glycine site-specific antagonist 5-nitro-6,7-dichloro-2,3-quinoxalinedione) is shown to counteract MDMA induced 5HT depletion and decrease in paroxetin binding [24]. NMDA receptors are possibly implicated in MDMA mediated mechanisms. However, the specific roles of glutamate and its receptors in response to MDMA administration are not well evaluated. In particular, knowledge of glutamatergic gene-transcript regulation following MDMA administration is limited.

The aim of the present study was to investigate the *immediate *effects of repeated intermittent MDMA administration upon gene-transcript levels of glutamatergic receptors and transporters. The mRNA levels of glutamate transporters (EAAT1, EAAT2-1, EAAT2-2), AMPA receptor subunits (GluR1, GluR2, GluR3), NMDA receptor subunits (NR1, NR2A and NR2B), as well as metabotropic glutamate receptors (mGluR1, mGluR2, mGluR3, mGluR5) were confirmed in six regions of the male rat brain (the prefrontal cortex (PFC), cortex, caudate putamen (CPU), nucleus accumbens (Acb), hippocampus and hypothalamus. These brain regions are known to be important for psychiatric conditions associated with MDMA abuse.

## Methods

### Animal treatment

Male adolescent Sprague-Dawley rats (weighing 216 ± 13 g and aged 7 weeks; Alab, Sollentuna, Sweden) were randomly separated into two MDMA ((±)-3,4-methylenedioxy-*N*-methamphetamine-HCl, Sigma Pharmaceutical)-treated groups and one control group. The rats were housed pair wise in air-conditioned rooms (12-h dark/light cycle) at 20°C and a humidity of 53%. All animals were subjected to one day of treatment every 7:th day for 4 weeks. On the drug-treatment day the animals received 3 intra-peritoneal injections given 3 hours apart (a binge); the MDMA low dose rats (n = 8) received 3 × 1 mg/kg MDMA, the MDMA high dose rats (n = 8) 3 × 5 mg/kg, whereas the controls received the vehicle of sterile 0.9% saline solution (1 ml/kg). The MDMA was dissolved into the vehicle on the day of testing. The rationale for these MDMA doses stems from human conditions, and has been presented elsewhere [25]. The performed experiments are approved by the local ethical committee in Stockholm, Sweden (N164/04) and are in agreement with international guidelines on the ethical use of animals. This ensures that the number of animals was minimized and the handlings monitored to avoid suffering. All rats were decapitated 10 h after the first injection of the last 4th MDMA binge to avoid withdrawal effects and study the immediate effects of repeated intermittent MDMA treatment.

Brain regions of interest (the prefrontal cortex (PFC), cortex at bregma +1.6 to +0.7 (cortex bregma +1.6) including cingulate cortex, primary motor cortex, and secondary motor cortex, caudate putamen (CPU), Acb (Acb), hippocampus (at bregma -4.8 to -5.6), and the hypothalamus (arcuate nucleus, ventromedial hypothalamus and partly the paraventricular nucleus) were dissected using a rat brain matrix [26], rapidly frozen on dry ice, immersed into RNA later (Ambion) for 1 hour, and then stored at – 80°C.

### Isolation of total RNA and reverse-transcription PCR

Total RNA was isolated from individual brain regions by phenol-chloroform extraction. Tissue samples (<100 mg) were homogenised in 500 μl TRIZOL (Invitrogen) by ultra sonication with a Branson sonifier. 100 μl chloroform was added. The homogenate was centrifuged at 10 000 rpm for 20 minutes. The RNA of the water-soluble supernatant was precipitated with isopropanol, and washed twice with ethanol, 75% and 80%, respectively. The air dried RNA pellet was dissolved in RNAase free water. DNAase (Roche) treatment was performed at 37°C for 3 hours in order to remove DNA contamination, followed by inactivation of the enzyme at 75°C for 15 minutes. The RNA purity was validated by polymerase chain reaction (PCR) and gel-electrophoresis using primers for a 300 bp cDNA of GAPDH (NM017008: tcc ctc aag att gtc agc aa; cac cac ctt ctt gat gtc atc). Total RNA concentration was determined using a nanodrop and was reverse transcribed using random primers, pd(N)^6^, according to manufacturers protocol (Amersham Biotech, Sweden). The cDNA synthesis was evaluated by PCR and gel-electrophoresis.

### Real Time PCR

The mRNA content of the various brain regions was assessed by real time PCR (quantitative PCR, qPCR) in terms of relative quantifications of amplified cDNA of interest using iCykler (Bio-Rad Laboratories, Sundbyberg, Sweden). The qPCR was performed in a final reaction volume of 20 μl. The concentration of templates consisting of synthesised cDNA was 1.25 ng/μl. The cDNA corresponding to transcripts of interest was identified by primers (see below) at a concentration of 0.8 pmol/μl for each of the sense and antisense, respectively.

In addition to template and primers, the qPCR reaction also contained 20 mM Tris/HCl (pH 8.4), 50 mM KCl, 4 mM MgCl_2_, 0.16 mM dNTP, SYBR Green (1:50,000), as well as Taq DNA polymerase (Invitrogen) at 0.02 U/μl. All qPCR experiments were performed in either triplicates or duplicates of each brain-tissue-cDNA template on an individual basis (n = 8/group). A negative control using water as template was included for each primer pair. For the qPCR protocol, annealing temperatures were between 58–62°C (depending on primer pair) and 50 cycles were used. Melting curves were included to confirm that only a single product was performed.

### Primers used for qPCR

Primers of used house-keeping genes (HKG) β-actin (ACT; NM_031144; cac tgc cgc atc ctc ttc ct; aac cgc tca ttg ccg ata gtg), cyclophilin (CYCLO; M19533; gag cgt ttt ggg tcc agg aat; aat gcc cgc aag tca aag aaa), glyceraldehyde-3-phosphate-dehydrogenase (GAPDH; X02231; aca tgc cgc ctg gag aaa cct; gcc cag gat gcc ctt tag tgg), histone H3b (H3b; NM_053985; att cgc aag ctc ccc ttt cag; tgg aag cgc agg tct gtt ttg), ribosomal protein-19 (RP19; NM:031103; tcg cca atg cca act ctc gtc; agc ccg gga atg gac agt cac), β-tubulin, beta 5 (TUB; NM_173102; cgg aag gag gcg gag agc; agg gtg ccc atg cca gag c), and succinate dehydrogenase complex A subunit A (SDHA; ggg agt gcc gtg gtg tca ttg; ttc gcc cat agc ccc cag tag) have previously been validated [27]. Primers of investigated gene transcripts of interest were designed with Beacon Designer (v2.1/v4.0) as follows: AMPA receptor subunits GluR1(NM_031608: caa cca ccg agg aag gat acc; ttc aca gtca acc acc acc ag), GluR2 (AF164344: ttg tga gga cta ccg cag aag; gga ctc cag caa gta ggc atac), GluR3 (NM_032990: gtt aca aat cac ggg cag ag tc; tgg cag gag cag gct taa ag); NMDA subunits NR1 (ctg caa ccc tca cttt tgag; tgc aaa agc cag ctg cat ct) (common to all splice variants) [28], NR2A (cag cag caa gcc aca gtt atg; agt ctc ggt agc cag gga ag), NR2B (caa gaa cat ggc caa cct gt; ggt aca cat tgc tgt cct tc) [28], metabotropic receptors mGluR1 (NM_017011: gcc acc aca cca cct ctg; tga cgg aat cag cca gga ac), mGluR2 (XM_001073809: ttg tgc gtg cct cac tcag; ctg tag gag cat cac tgt gg), mGluR3 (atg gtg tcc gtg tgg ctt atc; tga ctg ttt ccc gct tct ctg), mGluR5 (NM_017012: tgt cca cca cca acc aac tg; gcc tcc act ctc tga atc cc); glutamate transporters EAAT1/GLAST (NM_019225:tct cct cta ctt cct ggt aac; agg gtg gca gaa ctt gag), EAAT2-1/Glt1a (NM_017215/AY069978: gag gaa gaa cct tgg aaa cg; gga agc ctg ttt aga gca tc), EAAT 2-2/Glt1b (NM_001035233: AF451299: ttg ctt gtt tca cca gat tcc; gcc atc aaa gtt ctg aca acc). The Basic Local Alignment Search Tool (BLAST) from the National Center for Biotechnology Information (NCBI) was applied to ensure that all amplified sequences shared no homology and were different from any other cDNA in the database.

### Evaluation of housekeeping genes and data analysis

The iCycler v3.0 Software for windows was used to analyse qPCR data, selected as cycle numbers, which for each plate-set-up both were normalized to the highest cDNA sample amount (lowest Ct value) and corrected according to a formula developed by Pfaffl and Co-workers of which the efficiency was calculated by means of the program LinReg [29]. Relative cycle numbers of investigated gene-transcript levels were normalized by normalization factors based on the inclusion of multiple HKGs as reference genes [30]. The approach of using multiple HKGs instead of one single ensures a much more accurate quantification of the content of gene transcript expression. A visual basic program, GeNorm (provided by Vandesompele) was applied to evaluate which unique set of HKGs that was the most stable in terms of a gene-stability measure for a given set of samples (cDNA batches of the current treatment profile including all three investigated groups) of each particular brain region. This methodology has been described elsewhere [25,30].

### Statistics

Statistical analyses were conducted with Statview (Statview v.5.0 for Windows). Shapiro-Wilk's test was applied to analyse the Gaussian distribution. One-way ANOVA and Fischer's PLSD test were used for statistical analyses of relative gene-transcript levels.

## Results

The set of HKGs with the most stable gene-stability value for a particular brain region have previously been presented [25]. For each brain region 7 HKGs were run by qPCR. In the PFC 5 HKGs were evaluated to be the most stable (GAPDH, RP19, SDHA, H3b, CYCLO), in the cortex 3 HKGs (β-actin, GAPDH, RP19), CPU (GAPDH, RP19, TUB, H3b), Acb (β-actin, GAPDH, TUB, SDHA, H3b, CYCLO), hippocampus (β-actin, RP19, TUB, H3b), amygdala (β-actin, GAPDH, RP19, TUB, SDHA, CYCLO), and hypothalamus (β-actin, GAPDH, TUB, SDHA, H3b, CYCLO) [25].

**Figure 1:**
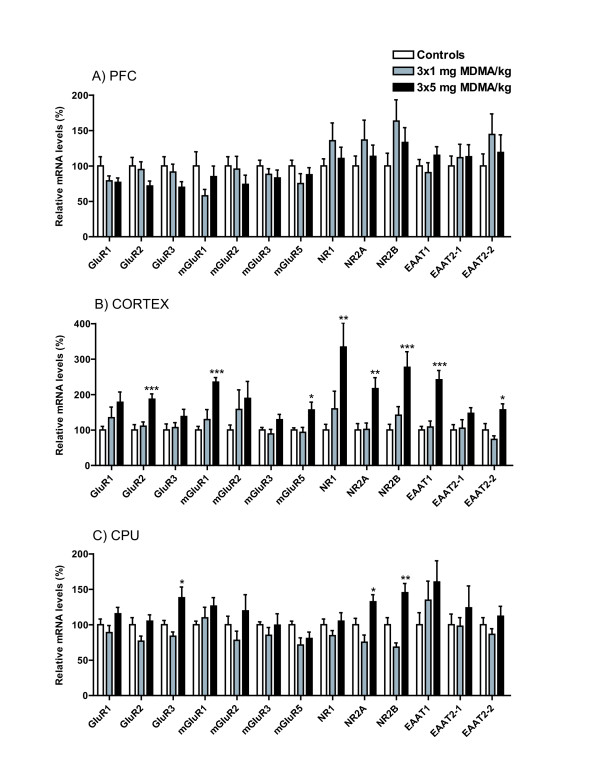
**Relative mRNA levels are presented in percent of relative cycle numbers for controls in response to repeated intermittent MDMA administration. A) PFC, B) Cortex, C) CPU**. *Statistical analysis: *Fischer's PLSD test was used for pair-wise analysis between independent groups in gene-transcripts where ANOVA analysis was considered to meet significance. Presented significance levels are related to control according to Fischer's PLSD: *P < 0.05, **P < 0.01, ***P < 0.001.

**Figure 2:**
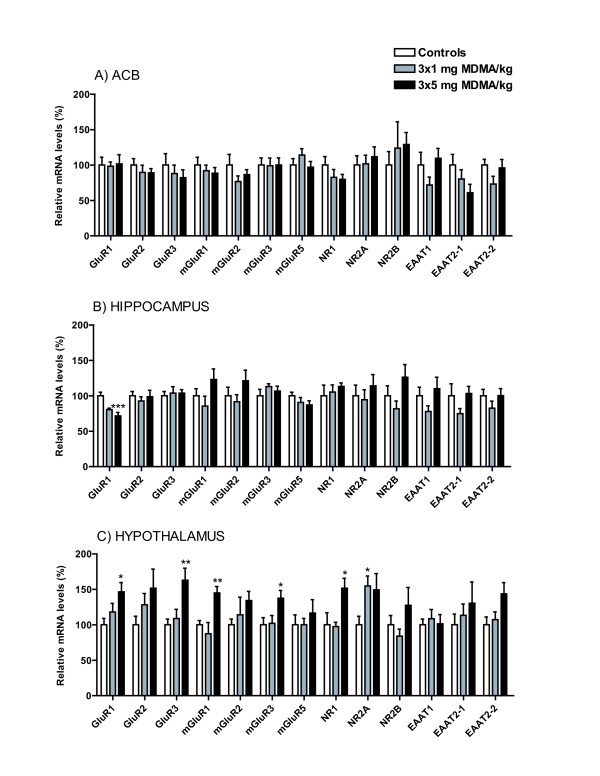
**Relative mRNA levels are presented in percent of relative cycle numbers for controls in response to repeated intermittent MDMA administration. A) Acb, B) Hippocampus, C) Hypothalamus**. *Statistical analysis: *Fischer's PLSD test was used for *pair-wise *analysis between independent groups in gene-transcripts where ANOVA analysis was considered to meet significance. Presented significance levels are related to control according to Fischer's PLSD: *P < 0.05, **P < 0.01, ***P < 0.001.

The effects of repeated intermittent MDMA administration, 3 × 1 mg/kg (MDMA low) or 3 × 5 mg/kg (MDMA high), every 7^th ^day for 4 weeks are presented as relative mRNA expressions in percent of the relative cycle numbers for controls, Figure 1 and 2. There were no significant alterations in the PFC, Figure 1A. The findings of ANOVA evaluated F-statistics and *P*-values presented as (mean ± SEM) for investigated mRNA levels in the PFC are as follows: GluR1 mRNA (F(2,20) = 1.05; P-value = 0.155): controls (100.0 ± 13), MDMA low (79.0 ± 7), and MDMA high (76.9 ± 6); GluR2 mRNA (F(2,21) = 2.08; P-value = 0.137): controls (100.0 ± 12), MDMA low (94.9 ± 11), and MDMA high (71.6 ± 7); GluR3 mRNA (F(2,20) = 2.10; P-value = 0.149): controls (100.0 ± 13), MDMA low (91.5 ± 11), and MDMA high (69.9 ± 8); mGluR1 mRNA (F(2,20) = 3.08; P-value = 0.201): controls (100.0 ± 20), MDMA low (57.9 ± 9), and MDMA high (84.9 ± 15); mGluR2 mRNA (F(2,17) = 1.03; P-value = 0.379): controls (100.0 ± 13), MDMA low (95.6 ± 18), and MDMA high (74.0 ± 13); mGluR3 mRNA (F(2,21) = 0.89; P-value = 0.740): controls (100.0 ± 8), MDMA low (88.1 ± 8), and MDMA high (83.2 ± 11); mGluR5 mRNA levels (F(2,20) = 1.33; P-value = 0.286); controls (100.0 ± 8), MDMA low (75.2 ± 14), and MDMA high (87.6 ± 10); NR1 mRNA (F(2,20) = 1.06; P-value = 0.365): controls (100.0 ± 10), MDMA low (135.7 ± 25), and MDMA high (110.5 ± 16); NR2A mRNA (F(2,19) = 0.82; P-value = 0.454): controls (100.0 ± 14), MDMA low (136.7 ± 28), and MDMA high (113.5 ± 16); NR2B mRNA (F(2,20) = 1.86; P-value = 0.181): controls (100.0 ± 18), MDMA low (163.4 ± 30), and MDMA high (133.1 ± 21); EAAT1 mRNA (F(2,19) = 1.17; P-value = 0.333): controls (100.0 ± 9), MDMA low (90.6 ± 14), and MDMA high (115.1 ± 12); EAAT2-1 mRNA (F(2,19) = 0.20; P-value = 0.817) controls (100.0 ± 14), MDMA low (111.7 ± 19), MDMA high (113.0 ± 17); EAAT2-2 mRNA (F(2,18) = 0.80; P-value = 0.463): controls (100.0 ± 17), MDMA low (144.5 ± 29), and MDMA high (119.0 ± 25).

In the cortex (bregma +1.6), MDMA high caused significant neuroadaptive enhancements of mRNA levels compared to controls for the AMPA subunit GluR2, the NMDA receptor subunits NR1, NR2A, and NR2B, the metabotropic receptors mGluR1 and mGluR5, as well as the glutamate transporters EAAT1 and EAAT2-2, Figure 1B. The findings of ANOVA analysis are as follows: GluR1 mRNA (F(2,15) = 1.93; P-value = 0.179): controls (100.0 ± 10), MDMA low (134.7 ± 30), and MDMA high (178.3 ± 29); GluR2 mRNA (F(2,15) = 11.73; P-value < 0.001): controls (100.0 ± 15), MDMA low (110.7 ± 12), and MDMA high (186.9 ± 15); GluR3 mRNA (F(2,20) = 0.15; P-value = 0.270): controls (100.0 ± 17), MDMA low (106.8 ± 14), and MDMA high (138.2 ± 20); mGluR1 mRNA (F(2,17) = 16.22; P-value < 0.001): controls (100.0 ± 10), MDMA low (129.5 ± 28), and MDMA high (235.0 ± 13), mGluR2 mRNA (F(2,18) = 1.40; P-value = 0.272): controls (100.0 ± 14), MDMA low (158.0 ± 55), and MDMA high (189.1 ± 48); mGluR3 mRNA (F(2,19) = 1.61; P-value = 0.073): controls (100.0 ± 7), MDMA low (88.8 ± 13), and MDMA high (129.0 ± 15); mGluR5 mRNA (F(2,18) = 5.72; P-value = 0.012): controls (100.0 ± 6), MDMA low (93.4 ± 14), and MDMA high (156.6 ± 22); NR1 mRNA (F(2,20) = 6.24; P-value = 0.008): controls (100.0 ± 16), MDMA low (159.5 ± 50), and MDMA high (334.0 ± 68); NR2A mRNA (F(2,17) = 8.08; P-value = 0.003): controls (100.0 ± 18), MDMA low (101.9 ± 18), and MDMA high (216.8 ± 31); NR2B mRNA (F(2,20) = 9.14; P-value = 0.002): controls (100.0 ± 16), MDMA low (141.8 ± 24), and MDMA high (276.8 ± 44); EAAT1 mRNA (F(2,18) = 19.0; P-value < 0.001): controls (100.0 ± 10), MDMA low (108.5 ± 17), and MDMA high (241.8 ± 26); EAAT2-1 mRNA (F(2,19) = 2.22; P-value = 0.136): controls (100.0 ± 15), MDMA low (105.2 ± 24), and MDMA high (147.0 ± 16); EAAT2-2 mRNA (F(2,19) = 6.71; P-value = 0.006): controls (100.0 ± 18), MDMA low (73.5 ± 10), and MDMA high (156.9 ± 17).

In the CPU, MDMA high significantly enhanced mRNA levels of the AMPA subunit GluR3, NMDA subunits NR2A and NR2B, Figure 1C. The findings are as follows: GluR1 mRNA (F(2,21) = 2.16; P-value = 0.140): controls (100.0 ± 8), MDMA low (88.9 ± 10), and MDMA high (115.5 ± 9); GluR2 mRNA (F(2,19) = 3.13; P-value = 0.067): controls (100.0 ± 10), MDMA low (76.9 ± 7), and MDMA high (105.1 ± 9); GluR3 mRNA (F(2,20) = 7.32; P-value = 0.004): controls (100.0 ± 6), MDMA low (83.8 ± 6), and MDMA high (138.2 ± 15); mGluR1 mRNA (F(2,17) = 1.01; P-value = 0.383): controls (100.0 ± 5), MDMA low (109.7 ± 15), and MDMA high (126.3 ± 12); mGluR2 mRNA (F(2,18) = 1.02; P-value = 0.382): controls (100.0 ± 12), MDMA low (78.0 ± 13), and MDMA high (119.5 ± 23); mGluR3 mRNA (F(2,19) = 0.59; P-value = 0.562): controls (100.0 ± 4), MDMA low (85.1 ± 11), and MDMA high (99.5 ± 16); mGluR5 mRNA (F(2,20) = 3.07; P-value = 0.061): controls (100.0 ± 5), MDMA low (71.4 ± 10), and MDMA high (80.6 ± 9); NR1 mRNA (F(2,20) = 1.31; P-value = 0.292): controls (100.0 ± 8), MDMA low (84.7 ± 7), and MDMA high (105.1 ± 12); NR2A mRNA (F(2,20) = 9.03; P-value = 0.002): controls (100.0 ± 9), MDMA low (75.4 ± 10), and MDMA high (132.5 ± 10); NR2B mRNA (F(2,17) = 13.59; P-value < 0.001): controls (100.0 ± 10), MDMA low (68.4 ± 6), and MDMA high (145.3 ± 13); EAAT1 mRNA (F(2,20) = 1.36; P-value = 0.285): controls (100.0 ± 17), MDMA low (134.6 ± 27), and MDMA high (160.3 ± 30); EAAT2-1 mRNA (F(2,19) = 0.42; P-value = 0.661): controls (100.0 ± 15), MDMA low (98.0 ± 12), and MDMA high (123.8 ± 31); EAAT2-2 mRNA (F(2,20) = 1.24; P-value = 0.310): controls (100.0 ± 10), MDMA low (86.4 ± 8), and MDMA high (112.0 ± 14).

In the Acb, there were no significant alterations observed, Figure 2A. Findings showed; GluR1 mRNA (F(2,19) = 0.40; P-value = 0.977): controls (100.0 ± 11), MDMA low (98.2 ± 6), and MDMA high (101.5 ± 13); GluR2 mRNA (F(2,19) = 0.98; P-value = 0.591): controls (100.0 ± 9), MDMA low (89.7 ± 10), and MDMA high (88.9 ± 6); GluR3 mRNA (F(2,19) = 0.47; P-value = 0.632): controls (100.0 ± 16), MDMA low (87.9 ± 12), and MDMA high (82.2 ± 11); mGluR1 mRNA (F(2,19) = 0.41; P-value = 0.672): controls (100.0 ± 11), MDMA low (91.9 ± 8), and MDMA high (88.5 ± 8); mGluR2 mRNA (F(2,19) = 1.16; P-value = 0.336): controls (100.0 ± 15), MDMA low (76.7 ± 8), and MDMA high (86.4 ± 7); mGluR3 mRNA (F(2,20) = 0.56; P-value = 0.997): controls (100.0 ± 10), MDMA low (98.9 ± 11), and MDMA high (100.0 ± 10) mGluR5 mRNA (F(2,19) = 1.12; P-value = 0.348): controls (100.0 ± 9), MDMA low (114.1 ± 9), and MDMA high (97.0 ± 8); NR1 mRNA (F(2,20) = 1.58; P-value = 0.317): controls (100.0 ± 12), MDMA low (82.7 ± 11), and MDMA high (79.8 ± 7); NR2A mRNA (F(2,19) = 1.01; P-value = 0.385): controls (100.0 ± 13), MDMA low (101.8 ± 12), and MDMA high (111.7 ± 14); NR2B mRNA (F(2,19) = 0.38; P-value = 0.727): controls (100.0 ± 19), MDMA low (123.8 ± 37), and MDMA high (129.0 ± 17); EAAT1 mRNA (F(2,19) = 1.57; P-value = 0.234): controls (100.0 ± 18), MDMA low (71.9 ± 11), and MDMA high (109.5 ± 14); EAAT2-1 mRNA (F(2,19) = 2.10; P-value = 0.150): controls (100.0 ± 15), MDMA low (80.3 ± 13), and MDMA high (60.8 ± 12); EAAT2-2 mRNA (F(2,20) = 1.85; P-value = 0.184): controls (100.0 ± 8), MDMA low (73.1 ± 11), and MDMA high (95.9 ± 12).

In the hippocampus, the AMPA subunit GluR1 mRNA was significantly decreased at the highest dose, Figure 2B. GluR1 mRNA (F(2,18) = 10.60; P-value < 0.001): controls (100.0 ± 5), MDMA low (80.4 ± 2), and MDMA high (71.5 ± 5); GluR2 mRNA (F(2,20) = 0.32; P-value = 0.730): controls (100.0 ± 6), MDMA low (92.6 ± 6), and MDMA high (98.8 ± 9); GluR3 mRNA (F(2,20) = 0.11; P-value = 0.900): controls (100.0 ± 6), MDMA low (103.8 ± 9), and MDMA high (103.8 ± 5); mGluR1 mRNA (F(2,20) = 1.84; P-value = 0.184): controls (100.0 ± 10), MDMA low (85.4 ± 14), and MDMA high (123.0 ± 15); mGluR2 mRNA (F(2,16) = 1.27; P-value = 0.308): controls (100.0 ± 12), MDMA low (91.6 ± 10), and MDMA high (121.1 ± 15); mGluR3 mRNA (F(2,19) = 0.69; P-value = 0.513): controls (100.0 ± 9), MDMA low (113.1 ± 4), and MDMA high (106.5 ± 7); mGluR5 mRNA (F(2,20) = 1.20; P-value = 0.323): controls (100.0 ± 5), MDMA low (90.6 ± 7), and MDMA high (86.9 ± 6); NR1 mRNA (F(2,19) = 0.34; P-value = 0.714): controls (100.0 ± 15), MDMA low (105.3 ± 10), and MDMA high (113.2 ± 5); NR2A mRNA (F(2,20) = 0.43; P-value = 0.655): controls (100.0 ± 15), MDMA low (94.5 ± 14), and MDMA high (114.0 ± 16); NR2B mRNA (F(2,19) = 2.05; P-value = 0.156): controls (100.0 ± 14), MDMA low (81.6 ± 11), and MDMA high (126.1 ± 18); EAAT1 mRNA (F(2,19) = 1.34; P-value = 0.284): controls (100.0 ± 12), MDMA low (77.8 ± 8), and MDMA high (110.1 ± 16); EAAT2-1 mRNA (F(2,20) = 1.47; P-value = 0.254): controls (100.0 ± 17), MDMA low (74.9 ± 7), and MDMA high (103.2 ± 10); EAAT2-2 mRNA (F(2,18) = 0.99; P-value = 0.391): controls (100.0 ± 9),, MDMA low (82.5 ± 10), and MDMA high (100.2 ± 10).

In the hypothalamus, the mRNA levels of GluR1, GluR3, mGluR1, mGluR3 and NR1 were significantly increased also after the administration of the highest MDMA dose, Figure 2C. In the same region, NR2A mRNA was significantly increased after repeated intermittent MDMA administration at a dose of 3 × 1 mg/kg, Figure 1D. GluR1 (F(2,20) = 3.92; P-value = 0.036): controls (100.0 ± 9), MDMA low (118.1 ± 12), and MDMA high (146.5 ± 13); GluR2 mRNA (F(2,21) = 1.54; P-value = 0.239): controls (100.0 ± 12), MDMA low (128.1 ± 16), and MDMA high (151.5 ± 27); GluR3 mRNA (F(2,19) = 6.43; P-value = 0.007): controls (100.0 ± 8), MDMA low (108.9 ± 13), and MDMA high (162.9 ± 17); mGluR1 mRNA (F(2,20) = 6.65; P-value = 0.006): controls (100.0 ± 6), MDMA low (87.3 ± 16), and MDMA high (144.9 ± 9); mGluR2 mRNA (F(2,16) = 1.12; P-value = 0.350): controls (100.0 ± 8), MDMA low (114.0 ± 25), and MDMA high (134.1 ± 13); mGluR3 mRNA (F(2,20) = 4.02; P-value = 0.034): controls (100.0 ± 10), MDMA low (102.0 ± 11), and MDMA high (137.4 ± 11); mGluR5 mRNA (F(2,21) = 0.41; P-value = 0.669): controls (100.0 ± 14), MDMA low (100.0 ± 9), and MDMA high (116.5 ± 19); NR1 mRNA (F(2,19) = 5.30; P-value = 0.015): controls (100.0 ± 17), MDMA low (97.5 ± 6), and MDMA high (151.5 ± 14); NR2A mRNA (F(2,18) = 3.21; P-value = 0.064): controls (100.0 ± 12), MDMA low (154.8 ± 14), and MDMA high (149.2 ± 23); NR2B mRNA (F(2,19) = 2.05; P-value = 0.156): controls (100.0 ± 13), MDMA low (83.9 ± 10), and MDMA high (127.5 ± 25); EAAT1 mRNA (F(2,21) = 0.15; P-value = 0.860): controls (100.0 ± 8), MDMA low (108.5 ± 13), and MDMA high (101.4 ± 13); EAAT2-1 mRNA (F(2,17) = 0.54; P-value = 0.594): controls (100.0 ± 15), MDMA low (113.4 ± 16), and MDMA high (130.4 ± 30); EAAT2-2 mRNA (F(2,18) = 3.12; P-value = 0.069): controls (100.0 ± 11), MDMA low (107.2 ± 11), and MDMA high (143.6 ± 16).

## Discussion

The present study provides evidence that repeated intermittent MDMA administration causes pronounced changes in mRNA expression of certain glutamatergic NMDA and AMPA receptor subunits, metabotropic receptors and transporters in the forebrain cortex, CPU and the hypothalamus. MDMA increased mRNA expression levels of glutamatergic NMDA receptor subunits NR1, NR2A and NR2B in the forebrain cortex, NR2A and 2B in the CPU, and NR1 and NR2A (lower dose) in the hypothalamus. Since NMDA receptor antagonists are protective for MDMA induced 5HT depletion and reduction of SERT density [22,24], the current shown MDMA induced enhancement in mRNA levels of glutamatergic NMDA receptor subunits may contribute to MDMA mediated neurotoxic effects. Furthermore, there are similarities between the immediate effects of long-term MDMA administration and other drugs of abuse. Repeated cocaine administration is shown to enhance NMDA receptor subunits in the VTA [31,32]. Chronic morphine administration is shown to increase NR1 mRNA levels in the hypothalamus [33]. Chronic alcohol administration is known to produce increased NMDA receptor subunits in response to suggested decreased glutamate levels [34-38]. Regarding glutamate transporters, EAAT1 (GLAST) and EAAT2-2 (Glt1b/EAAT2b) (splice variant of EAAT2/Glt1) were significantly increased at the highest administered MDMA dose in the cortex. These glutamate transporters play important roles in order to maintain extracellular glutamate levels, mainly by being expressed on glial astrocytes [39,40]. Overactive glutamate transporters may cause low glutamate levels. Taken together findings of mRNA expression levels, both in NMDA receptor subnunits and glutamate transporters, suggest low glutamate levels in response to repeated intermittent MDMA administration.

Corticostriatal glutamatergic neurons projecting from the forebrain cortex to sub-cortical brain regions in the CPU and Acb regions are involved in the pathophysiology of drug addiction and schizophrenia [41,42]. Reduced glutamatergic activity in this pathway is associated with psychotic symptoms from drug abuse and schizophrenia [43,44]. Interestingly, chronic haloperidol treatment down regulate EAAT2 in the cingulate- and frontal cortex in rats [45], indicating that decreased EAAT2 levels rather may reflect a beneficial condition following the antipsychotic treatment of schizophrenia. MDMA induced enhancements in the EAAT 2-2 mRNA level in the forebrain cortex may therefore partly contribute to psychotic effects of this drug.

Glutamatergic mRNA expression of AMPA receptor subunits was also affected by repeated intermittent MDMA administration. The mRNA level of GluR2 was increased in the forebrain cortex, GluR3 in the CPU, and GluR1 and GluR3 in the hypothalamus. In the forebrain cortex, glutamate mediated activation of AMPA-KA receptors, either localised on 5-HT nerve terminals or cortifugal glutamatergic pyramidal neurons projecting to the dorsal raphe nucleus, is in turn suggested to enhance 5HT release *via *stimulation of 5HT_2A _receptors [46]. Hallucinogenic drugs such as DOI and LSD are interestingly suggested to activate 5HT_2_-receptors on GABA neurons in the cortex with a strengthened inhibitory effect upon cortiostriatal glutamate neurons to follow. This view is in agreement with the fact that the initial MDMA mediated increase of dopamine- and serotonin neurotransmission [21], inhibits glutamate evoked firing in the nucleus accumbens [20]. In our previous study, the 5HT_2A _receptor mRNA was significantly reduced, whereas the 5HT_2C _receptor mRNA was enhanced after repeated intermittent MDMA administration [25], indicating on compensatory mechanisms in response to long term MDMA administration. Findings of our current study may explain psychotic and hallucinogenic symptoms from repeated intermittent MDMA administration. However, some corticofugal glutamate pathways are implicated circuits accelerating and other in breaking the activity of subcortical systems in sophisticated interplay [13].

In the hippocampus GluR1 mRNA was significantly decreased. This brain region is important for the regulation of cognitive functions and memory. Interestingly, GluR1 is known to be implicated in memory functions [47] and may play a role in MDMA induced cognitive deficits [2,48,49]. Memory loss in response to MDMA is previously reported to be caused by prevented learning specific increase in NMDA NR1 expression and Ca2+/calmodulin-dependent protein kinase II (CaMKII) posphorylation by reduced scaffolding postsynaptic density protein PSD-95 levels, instead of direct effects by reduced 5HT activity itself [50,51]. However, in the hippocampus repeated intermittent MDMA administration did not cause any changes in mRNA levels of serotonin receptors [25], nor NMDA subunits, indicating that other mechanisms, here, primarily underlie potential MDMA elicited memory deficits. This is additional support for the diversity of studies on MDMA induced memory dysfunctions [52].

In the hypothalamus, metabotropic mGluR1 and mGluR3 receptors were also increased in addition to previously described findings of elevated mRNA levels in glutamatergic AMPA and NMDA subunits. Neuroadaptive alterations in the hypothalamus could reflect involvement of numerous different mechanisms regulating neurendocrine functions, as well as motivational drives towards feeding behaviour and rewarding properties of the corticostriatal-hypothalamic-brainstem network [53-55].

There were no significant changes in any of the investigated glutamatergic gene-transcipts in the Acb in response to repeated intermittent MDMA administration. Since the Acb is an interactive target between glutamatergic cortico-striatal projections and mesolimbic dopamine neurons [14], and well known to be implicated in compulsive drug abuse [56,57], this finding is very interesting. Although MDMA causes an immediate increase in dopamine release in the Acb [58,59], it is suggested to possess a primary action upon the serotonergic system in rats [9,25,60]. One potential explanation to the absence in glutamatergic mRNA changes in response to MDMA in the Acb might therefore be that interactive processes between serotonergic and glutamatergic systems, here, are less pronounced than between dopaminergic and glutamatergic systems. On the other hand, taking into account that mRNA expression of serotonin receptors also is reported to be more extensive in the cortex than other brain areas in response to repeated intermittent MDMA administration [25], it is tempting to hypothesize that MDMA rather is implicated in cognitive, associative, and hallucinogenic responses than reward-related craving or incentive salience attribution [61,62]. Furthermore, future studies have to achieve increased knowledge of the glutamatergic interactions with the GABA and serotonergic activites in the forebrain cortex [13], in order to better understand MDMA induced responses. Furthermore, the posttranscriptional roles of glutamate-receptor subunits and glutamate transporters also have to be fully evaluated.

## Conclusion

Pronounced alterations of mRNA expression in glutamate transporters, AMPA and NMDA receptor subunits in regions of the adolescent male rat brain may contribute to deficits in cognitions, memory and neuroendocrine functions as well as hallucinations and psychosis from repeated intermittent MDMA administrations.

## Authors' contributions

AKH conceived, designed and carried out the study and wrote the manuscript. AB and RM performed and analysed the real time PCR-experiments. HBS participated in the manuscript writing. All authors read and approved the final manuscript.
